# Unusual Disease‐Progression in Two Siblings With Xeroderma Pigmentosum Group G

**DOI:** 10.1111/cge.70129

**Published:** 2026-01-02

**Authors:** Elena Botta, Heather Fawcett, Donata Orioli, Hiva Fassihi, Alan R. Lehmann

**Affiliations:** ^1^ Institute of Molecular Genetics‐CNR Pavia Italy; ^2^ Genome Damage and Stability Centre University of Sussex Brighton UK; ^3^ National Xeroderma Pigmentosum Service, GSTT‐NHS Foundation Trust London UK

## Abstract

Protein truncation mutations in the gene for XPG nuclease cause a very severe clinical phenotype. Two siblings have splicing mutations, which result in in‐frame deletions and a less severe phenotype.

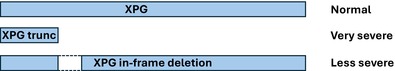

1

Xeroderma pigmentosum (XP) is a recessive disorder characterized by hypersensitivity to ultraviolet radiation (UVR)‐induced lentigines and skin cancers, and in some cases by associated neurological abnormalities [1, 2]. It results from mutations in one of nine genes, eight of which (*XPA* to *XPG* and *XPJ*) encode proteins involved in different steps of nucleotide excision repair (NER) of bulky DNA damage.

XPG protein is a 3′‐flap endonuclease that, in NER, cleaves the DNA strand 3′ to the damage. The active site of the protein contains the N and I domains separated by a long unstructured spacer region likely involved in different protein–protein interactions, as XPG has several other roles [3]. Mis‐sense mutations in the active site regions result in XP patients with varying neurological abnormalities. However, protein truncations usually result in a more severe phenotype with XP features combined with neurodegeneration typical of Cockayne syndrome (CS) and death in early childhood [3].

Patients XP56BR, a 28‐year‐old male, and XP55BR, a 25‐year‐old female, are siblings born in Somalia, who moved to the UK in early childhood. The first clinical features of XP were severe and exaggerated sunburn, photophobia, and facial lentigines from 6 months of age. They have short stature, severe sensorineural hearing loss, neurological impairment, poor cognition and moderate learning difficulties, but no skin cancers. Nerve conduction is normal. Magnetic resonance imaging (MRI) of the brain shows central white matter volume loss. They have relative enophthalmos. These clinical features indicate XP/CS overlap. The short stature, cachexia in the brother, enophthalmos and the MRI findings, are typical of CS. The severe sunburn reactions and lentigines are characteristic of XP. Unusually for XPs with neurodegeneration, they remain relatively mobile and have no peripheral neuropathy.

Skin fibroblast cultures were established and found to be NER‐defective after UVR (Figure 1A). As previously described, molecular analysis identified a homozygous deletion of the invariant G at the first base of intron 2 (c.264 + 1delG), which is expected to result in abnormally spliced mRNA. This mutation at aa88, in the N‐domain of the XPG nuclease active site (Figure 1B), accounts for the undetectable NER in patients' cells. A truncation at this position (aa 88) would be expected to result in a very severe XP‐CS phenotype. However, quantitative RT‐PCR, using primers indicated in Figure 1C,D, revealed that in patients' cells the total *ERCC5/XPG* transcript level was approximately 50% of that in the normal control (Figure 1E). About 90% of the mutated transcripts are missing exon 3 (nucleotides 264–380; Figure 1D), resulting in 39 aa *in‐frame* deletion, whereas about 10% are missing exon 2 and part of exon 1 (nucleotides 48‐263; Figure 1D), resulting in 72 aa *in‐frame* deletion. Though inactivating the nuclease active site, both deletions leave the rest of the protein intact and available for protein–protein interactions involved in other roles of the XPG protein [3]. Using immunoblots to detect XPG protein (Figure 1F), a faint band of the expected size was found in control but not in patients' extracts. Smaller bands corresponding to the expected *in‐frame* deletions could not be identified in the patients' extracts, indicating that, if present, the amount of mutated XPG variants is below the detectable level. Nevertheless, we propose that this is sufficient to delay the onset of the most severe features. Our studies emphasize the importance of detailed analysis of splice‐site mutations to understand clinical phenotypes. *In‐frame* deletions or alternative initiation‐sites may generate products with residual function. Alternatively, a small amount of read‐through from the splice‐site may result in residual levels of normal protein. In any of these scenarios, the clinical features of affected individuals may be less severe than those of individuals with null mutations.

**FIGURE 1:**
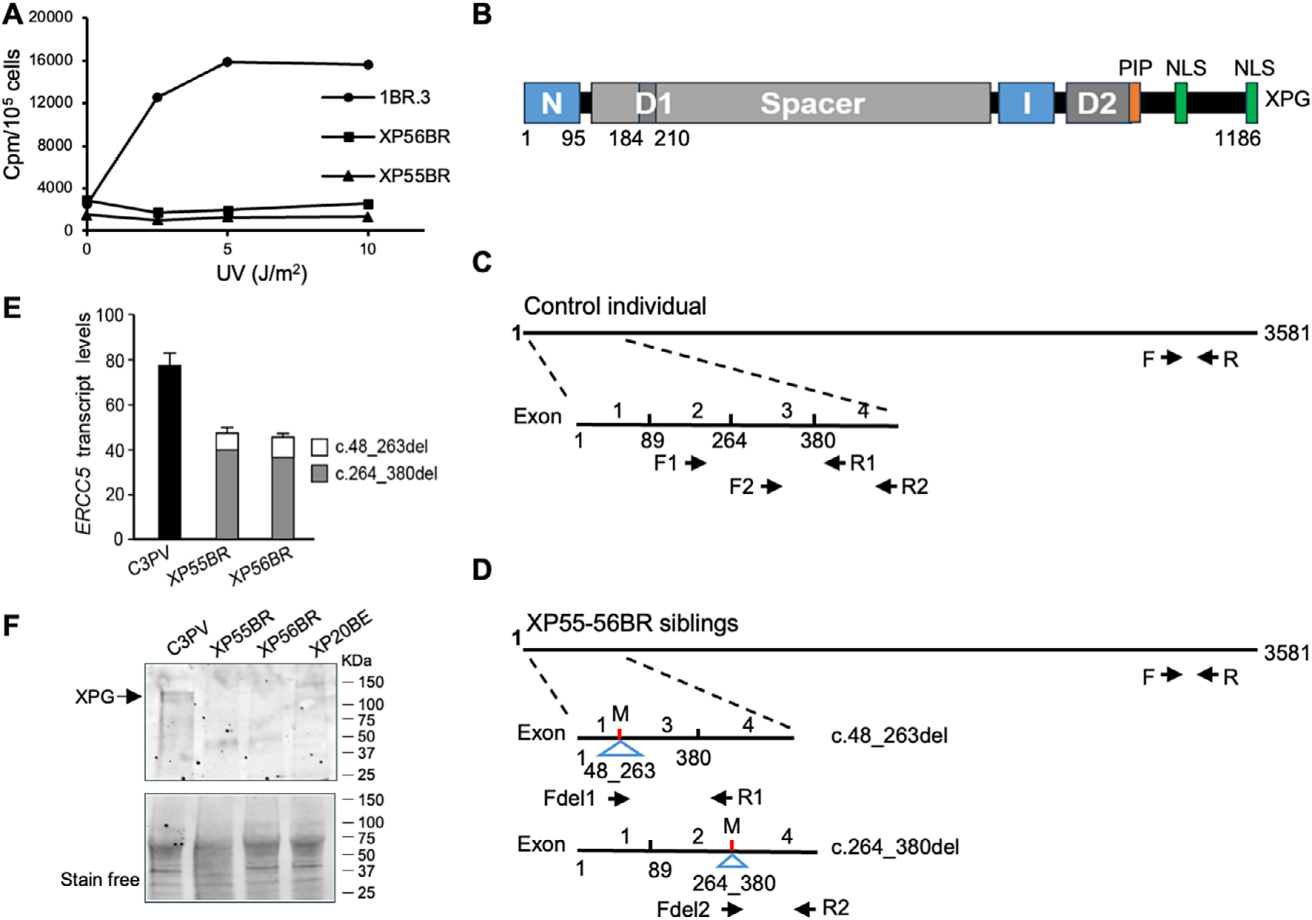
(A) Unscheduled DNA Synthesis in control (1BR.3) and patients' (XP55BR‐XP56BR) cells. (B) Schematic of the XPG protein. *ERCC5/XPG* cDNA expressed by normal cells (C) or XP55‐56BR cells (D). Positions of primers used in RT‐PCR for determining the amount of *ERCC5/XPG* transcripts are indicated. M, position of mutation. (E) Amount of *ERCC5/XPG* transcripts using primers indicated in (C) and (D). C3PV, normal control. (F) Immunoblot of whole‐cell lysates probed with anti‐XPG antibody. XP20BE, XP‐G cells with null mutation.

## Ethics Statement

This study was performed in accordance with protocols approved by the Research Ethics Committee of GSTT‐NHS Foundation Trust, London (reference 12/LO/0325).

## Consent

Informed consent was obtained from the patients' family for use of clinical data.

## Conflicts of Interest

The authors declare no conflicts of interest.

## Data Availability

Data can be obtained from the authors on request.
